# Clusterin Deficiency Promotes Cellular Senescence in Human Astrocytes

**DOI:** 10.1007/s12035-024-04650-2

**Published:** 2024-12-03

**Authors:** Pinky Sultana, Ondrej Honc, Zdenek Hodny, Jiri Novotny

**Affiliations:** 1 Department of Physiology, Faculty of Science, Charles University, Prague, 128 00 Czech Republic; 2 Laboratory of Genome Integrity, Institute of Molecular Genetics of the Czech Academy of Sciences, Prague, 142 20 Czech Republic

**Keywords:** Astrocytes, Cellular senescence, Clusterin, Oxidative stress, Mitochondria

## Abstract

The glycoprotein clusterin (CLU) is involved in cell proliferation and DNA damage repair and is highly expressed in tumor cells. Here, we aimed to investigate the effects of CLU dysregulation on two human astrocytic cell lines: CCF-STTG1 astrocytoma cells and SV-40 immortalized normal human astrocytes. We observed that suppression of CLU expression by RNA interference inhibited cell proliferation, triggered the DNA damage response, and resulted in cellular senescence in both cell types tested. To further investigate the underlying mechanism behind these changes, we measured reactive oxygen species, assessed mitochondrial function, and determined selected markers of the senescence-associated secretory phenotype. Our results suggest that CLU deficiency triggers oxidative stress–mediated cellular senescence associated with pronounced alterations in mitochondrial membrane potential, mitochondrial mass, and expression levels of OXPHOS complex I, II, III and IV, indicating mitochondrial dysfunction. This report shows the important role of CLU in cell cycle maintenance in astrocytes. Based on these data, targeting CLU may serve as a potential therapeutic approach valuable for treating gliomas.

**Supplementary Information:**

The online version contains supplementary material available at 10.1007/s12035-024-04650-2.

## Introduction

Astrocytes play an essential role in many aspects of brain physiology, and the loss of their function or the adoption of a neuroinflammatory phenotype associated with cellular senescence may contribute to the pathology of age‐related neurological diseases [1]. Various forms of senescence often result from pathological stressors such as oncogene activation, oxidative stress, mitochondrial dysfunction, chemotherapy, or radiation exposure [2]. In general, cellular senescence plays a role in fundamental biological processes such as embryogenesis and tissue regeneration. It is also considered a fundamental protective mechanism against tumorigenesis. A hallmark of senescence is an essentially irreversible interruption of cell division due to the stress factors mentioned above, resulting in persistent DNA damage response and activation of the cell cycle checkpoints. Persistent DNA damage, occurring mainly at specific genomic loci, such as telomeres or ribosomal DNA, is the most frequent cause of senescence development. This damage, in the form of unhealed DNA double-strand breaks (DSBs), permanently activates the DNA damage response (DDR) pathway via the ATM/p53/p21WAF1 axis, resulting in cell cycle arrest mediated by p21WAF1 (p21), which is enhanced by the expression of another cyclin-dependent kinase inhibitor, p16INK4A (p16). The coordinated action of p21 and p16 is responsible for long-term cell cycle arrest and the development of the senescent phenotype [3, 4]. Despite the primary preventive role of senescence induction in cancer promotion, the accumulation of senescent cells during aging contributes to tissue dysfunction and age-related diseases. The senescence-associated secretory phenotype (SASP) [5], which is triggered by oxidative stress and DNA damage, is considered one of the most important pathological factors contributing to age-related tissue dysfunction, such as impaired tissue regeneration, supporting the malignant properties of cancer cells and inducing mild but chronic inflammation in tissues through the release of pro-inflammatory factors [6].

Clusterin (CLU), or ApoJ, is a glycoprotein with two isoforms: secreted (sCLU) and nuclear (nCLU) clusterin [7]. CLU is important for cell proliferation and cell cycle regulation, DNA repair signaling pathways, membrane recycling, and immune system regulation [8, 9]. In the brain, astrocytes are the main source of CLU. However, CLU expression has also been observed in a subpopulation of neurons [10]. CLU is frequently upregulated in various human cancers and contributes significantly to tumor progression, chemoresistance, and metastasis [11]. In human colon cancer, for example, overexpression of CLU has been associated with tumorigenesis and progression to metastasis [12]. In contrast, the knockdown of CLU in pancreatic cancer cells limited tumor progression by inhibiting the NF-κB signaling pathway [13]. It has been reported that the sensitivity of cancer stem cells to the chemotherapeutic agent camptothecin was increased when the cells were treated with a combination therapy that included silencing of CLU [14]. Furthermore, the silencing of CLU in human prostate carcinoma PC3 cells remarkably sensitized them to the anticancer drug paclitaxel [15]. Interestingly, some preclinical attempts have been made to use an innovative phosphorothioate antisense oligonucleotide compound custirsen (OGX-011), which targets and regulates the expression of sCLU, for the treatment of metastatic castration–resistant prostate cancer in combination with docetaxel or mitoxantrone [16]. In parallel, several cancer studies have shown that silencing CLU leads to cell proliferation arrest, for example, in renal cell carcinomas [17], human breast cancer cells [18], lung cancer [19], and osteosarcoma [20]. Recently, it was observed that CLU silencing induces cellular senescence in pancreatic cancer cells [21].

It is generally recognized that astrocyte senescence can have a significant impact on the progression of neurodegenerative diseases. Aging biomarkers such as p16 have been found to be elevated in aged cortices, and p16 is overexpressed in the frontal cortex of Alzheimer’s disease patients [22]. Several studies have linked astrocyte senescence to oxidative stress and mitochondrial dysfunction [23, 24]. Excessive production of reactive oxidative species (ROS) and antioxidant deficiency may trigger cellular senescence due to oxidative stress [25]. Astrocytes in a healthy brain release CLU into the extracellular space. However, CLU expression in astrocytes and neurons is increased during inflammation, stress, or neurodegenerative diseases [26]. Today, it is not clear if and how CLU deficiency might affect astrocyte viability and survival.

In the present study, we investigated the consequences of CLU downregulation in human astrocytes and described the molecular changes associated with siRNA silencing–mediated CLU dysregulation in CCF-STTG1 astrocytoma and SV-40 immortalized non-transformed human astrocyte cell lines. We observed that suppressing CLU can induce cellular senescence in astrocytes, preceded by increased ROS production and alterations in mitochondrial function.

## Materials and Methods

### Cell Lines and Culture Conditions

The human brain astrocytoma cell line CCF-STTG1 (CCF, obtained from the European Collection of Authenticated Cell Cultures) and SV40-immortalized non-transformed human astrocytes (INHA, obtained from Innoprot, Spain) were grown in DMEM (Sigma-Aldrich, St. Louis, MO, USA) with 10% fetal bovine serum, 100 U/ml penicillin, and 100 μg/ml streptomycin (Sigma-Aldrich, St. Louis, MO, USA), at 37 °C in a humidified atmosphere of 5% CO_2_.

### Small Interfering RNA Reverse Transfection

Cells at a density of 100,000 cells per well in a 6-well microplate were transfected with 25 nM CLU siRNA (siCLU, Cruz Biotechnology Biotechnology; sc-43688) or non-targeting siRNA (siNC, Ambion; #4390843) using Lipofectamine RNAiMAX transfection reagent (Invitrogen, Carlsbad, CA, USA) according to the manufacturer’s protocol. Incubation of cells in the presence of the transfection mixture with siRNA lasted for 72 h. Subsequently, cells were used for experiments.

### Crystal Violet Assay

The crystal violet assay was used as a screening method to investigate the impact of CLU downregulation on cells [27]. CCF and INHA cells at a density of 60,000 cells per well in a 12-well plate were reverse transfected with siCLU or siNC. Three days after transfection, cells were fixed with methanol at 4 °C for 20 min, followed by crystal violet staining (0.5% crystal violet solution in 25% methanol) for 10 min to examine growth arrest.

### Automated Cell Counting

Cells transfected with CLU siRNA or non-targeting siRNA were cultured for up to 72 h. Cell imaging was conducted up to 10 days using the BioTek Cytation 5 cell imaging multimode reader (Agilent) at 4 × magnification. Automated cell counting was performed using Gen5 software integrated with the Cytation 5 system (Agilent Technologies, Inc.). Cell growth quantification was based on data from three independent experiments.

### Cell Proliferation Assay

The colorimetric MTT assay was used to determine cellular metabolic activity to indicate cell viability and proliferation. Cells were seeded at a density of 7500 per well in a 96-well plate. After transfection of cells with siCLU or siNC, an MTT assay was performed as described previously [28]. The cells were monitored for up to 72 h.

### Scratch Wound Migration Assay

For the scratch wound assay, 60,000 cells were reverse-transfected and seeded in 12-well plates. After 2 h, the monolayer was scratched using a 1-mm pipette tip. The cells were then further incubated for 24 to 72 h. The images of the scratched cell monolayers were taken with a phase contrast microscope at 4 × magnification. The width of the scratches was measured using ImageJ, and the speed of cell migration was calculated from three individual experiments.

### Assessment of Cell Cycle Using Flow Cytometry

Cells were fixed in 70% cold ethanol and stored at − 20 °C for 30 min. They were then stained with DAPI (Sigma-Aldrich; D9542) staining solution (1 mg/ml DMSO) at room temperature for 5 min. The cells stained with DAPI were analyzed for DNA content by flow cytometry using BD LSR II flow cytometer (BD Biosciences, San Jose, CA, USA), and the data were evaluated using KALUZA software.

### Cytochemical Assay of Senescence-associated β-Galactosidase

Senescence-associated β-galactosidase (SA-β-Gal) staining was performed using an SA-β-Gal Staining Kit (Sigma-Aldrich) according to the manufacturer’s instructions. CCF and INHA cells were reverse transfected with siCLU for 72 h. After treatment, cells were washed in phosphate-buffered saline (PBS) and stained for 16 h according to the kit instructions. The blue cells were imaged under a light microscope (Leica DMI 3000B, Solms, Germany) and counted.

### Immunofluorescence Staining

Briefly, cells were fixed with 4% formaldehyde at room temperature for 10 min, then permeabilized with 0.1% Triton X-100 for 3 min and blocked with 3% bovine serum albumin (BSA) at room temperature for 1 h. Cells were incubated with primary antibodies against CLU, γH2AX, or Ki67 (1:200) for 90 min. After washing, they were incubated with secondary Alexa Fluor 568-conjugated goat anti-rabbit IgG or Alexa Fluor 488-conjugated goat anti-mouse IgG (1:1000) antibodies for 60 min. To visualize nuclei, the specimens were simultaneously stained with DAPI. Finally, the images were captured with a Leica DM 6000 microscope (Solms, Germany).

### Determination of Intracellular Reactive Oxygen Species

Intracellular reactive oxygen species (ROS) were measured with 2,7-dichlorofluorescein diacetate (DCF-DA, Sigma-Aldrich). Briefly, CCF and INHA cells were reverse transfected with 25 nM siCLU at a density of 10,000 cells/cm^2^ in a 96-well plate and cultured for 72 h. After washing once with PBS, cells were incubated with 10 µmol DCF-DA at 37 °C in the dark for 30 min, washed several times with PBS, and imaged with a fluorescence microscope (AIF 5013i-T, Arsenal, Czechia). Fluorescence intensity was measured with a microplate reader at 495/527 nm (ex/em). Digital images were acquired randomly. Data were evaluated using Gen software. For flow cytometry, 200,000 cells were seeded in 12-well plates and cultured for 72 h with and without siCLU transfection. The cells were then trypsinized and centrifuged for flow cytometry analysis using BD LSR II flow cytometer, and the data were analyzed using KALUZA software.

### Measurement of Mitochondrial Membrane Potential

Mitochondrial membrane potential evaluation was assessed using the TMRE (tetramethylrhodamine ethyl ester) fluorescent probe. CCF and INHA cells plated in 96-well plates (black side, transparent bottom) were reverse transfected with siCLU for 72 h. Cells were loaded with 100 nM TMRE during the last 20 min of incubation. FCCP (trifluoromethoxy carbonylcyanide phenylhydrazone), a potent uncoupler of oxidative phosphorylation in mitochondria, was used as a positive control, and cells were incubated in the presence of 1 μM FCCP for 5 min. Cells were washed with PBS and resuspended in 0.2% BSA in PBS. Fluorescence intensity at 549/575 nm (ex/em) was determined using a microplate reader (BioTek synergy HT, Biotek Instruments, Winooski, VT, USA). Fluorescence images were captured with a fluorescent microscope (AIF 5013i-T). Data were evaluated using Gen software. For flow cytometry, 200,000 cells were seeded in 12-well plates and cultured for 72 h with and without siCLU transfection. The cells were then trypsinized and centrifuged for flow cytometry analysis (BD LSR II flow cytometer), and the data were analyzed using KALUZA software.

### Assessment of Mitochondrial Mass

Mitochondrial mass was measured in cells loaded with MitoTracker Green (Invitrogen). Briefly, after transfection, CCF and INHA cells grown in plates were incubated with 100 nM MitoTracker Green at 37 °C for 30 min. Images were then acquired with a fluorescent microscope (AIF 5013i-T), and fluorescence intensity was measured using a microplate reader at 492/515 nm (ex/em) and data normalized to Hoechst33342 staining.

### Determination of the ATP Production Rate

CCF and INHA cells were seeded at a density of 7500 in Seahorse XF 8-well poly-D-lysine-coated plates and after transfection with siNC (control) or siCLU cultured for 3 days. Mitochondrial and glycolytic ATP production rates were measured according to the Agilent Seahorse Real-Time Cell Metabolic Analysis protocol and analyzed using Agilent Seahorse Analytics software.

### Protein Determination

Protein concentration was determined by the bicinchoninic acid (BCA) assay. Samples were incubated in the presence of copper ions and BCA in an alkaline environment for 30 min at 37 °C, followed by absorbance measurement at 562 nm with a microplate reader.

### Western Blotting Analysis

Western blotting analysis was performed as previously described [29]. Briefly, the cells were lysed in RIPA lysis buffer containing a protease inhibitor cocktail (Sigma-Aldrich). The lysates (equal amounts of protein) and Laemmli buffer (4 × concentrated) were then mixed and boiled for 2 min before loading onto the gel (Bio-Rad, Hercules, CA, USA). Proteins were separated under constant voltage (200 V), and then transferred electrophoretically to 0.45 μM or 0.2 μM nitrocellulose membranes (Trans-Blot Turbo; Bio-Rad). The membranes were then blocked in 5% BSA at room temperature for 1 h and incubated at 4 °C overnight with primary antibodies. After the blots were washed three times with TBS containing 0.1% Tween-20 (wash buffer), they were incubated with a horseradish peroxidase-linked secondary antibody (anti-mouse/anti-rabbit/anti-goat immunoglobulin G; Santa Cruz Biotechnology, Inc., Dallas, TX, USA) at room temperature for 1 h. Membranes were then washed three times with a wash buffer. FEMTO chemiluminescent substrate was used to develop the blots according to the manufacturer’s instructions (Pierce Biotechnology, Rockford, IL, USA). Blots were scanned and quantitatively analyzed using ImageJ software. Data were normalized to Ponceau S or beta-actin staining and expressed as fold change relative to the loading control.

### Statistical Analysis

All experiments were performed in at least three independent biological replicates. Statistical analyses were performed using GraphPad Prism software version 8.0 (GraphPad Software, San Diego, CA, USA). All data were expressed as mean ± SEM (standard error of the mean). The differences between the groups were analyzed using one-way ANOVA, and *p*-values below 0.05 were considered statistically significant.

## Results

### Downregulation of CLU Suppresses Proliferation and Migration of Astrocytes

**Fig. 1:**
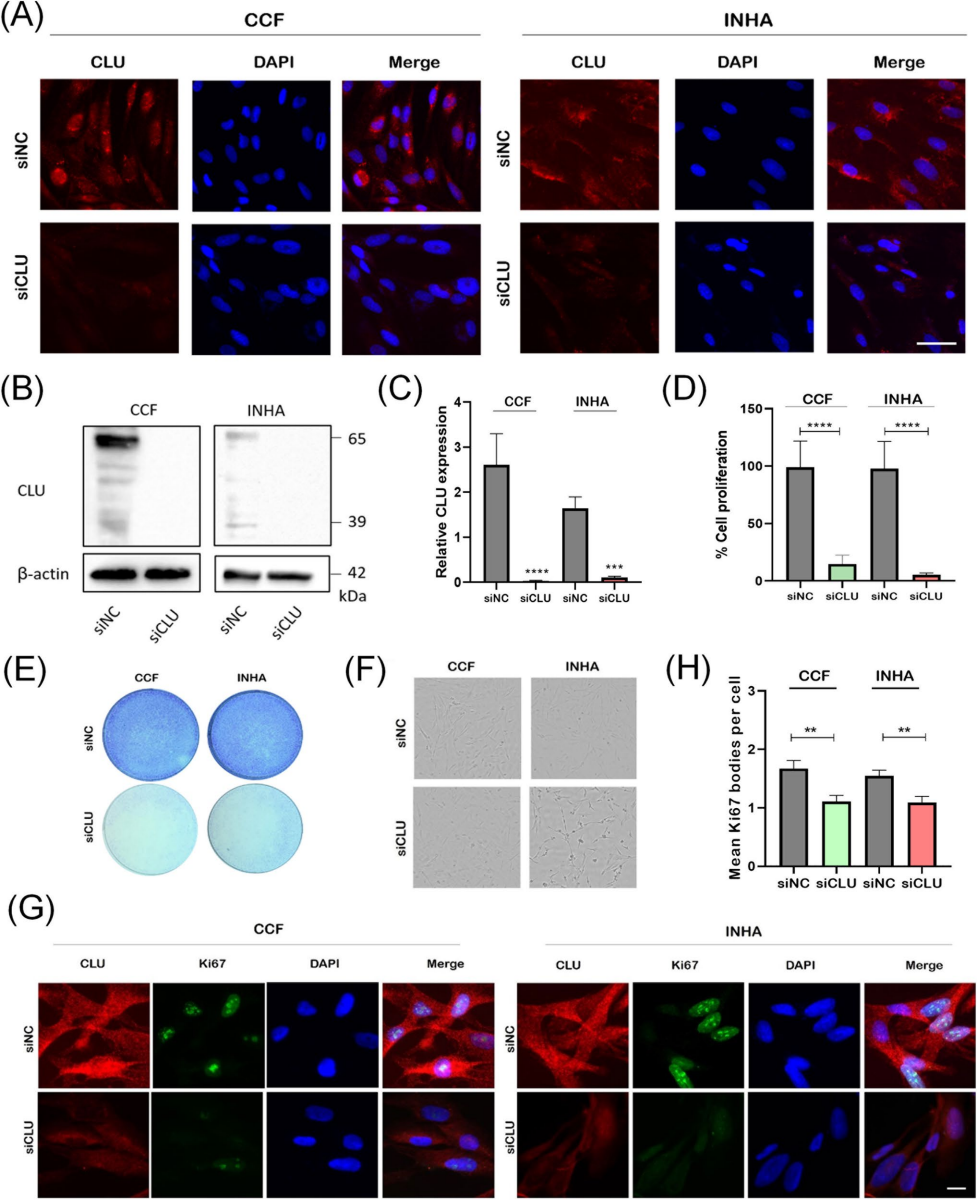
Downregulation of CLU inhibits proliferation of human astrocytes. Representative examples of fluorescence microscopy images (20 × magnification) of control (siNC) and CLU-silenced (siCLU) CCF-STTG1 astrocytoma cells (CCF) and immortalized non-transformed human astrocytes (INHA) labeled with a primary antibody against CLU and Alexa Fluor 568-conjugated secondary antibody (red emission). In addition, cells were simultaneously stained with DAPI (blue emission) (**A**). Scale bar, 100 µm. Representative Western blots show the expression levels of CLU in homogenates of CCF and INHA cells transfected with siNC or siCLU (**B**). Quantitative analysis of CLU expression using Western blotting (**C**). Effect on cell growth induced in CCF and INHA cells by transfection with siCLU as determined by MTT assay 72 h after transfection (**D**). Crystal violet staining indicates growth arrest in CLU-silenced (siCLU) cells compared with control (siNC) cells (**E**). Bright-field micrographs provide visual evidence of the morphological differences between control and CLU-deficient cells (**F**). Ki-67 staining in siNC and siCLU cells (**G**) shows a reduced number of Ki-67 bodies after CLU downregulation (**H**), scale bar, 20 µm. Values represent the mean ± SEM of three independent experiments (**p < 0.01; ***p < 0.001; ****p < 0.0001)

To investigate the effect of CLU knockdown on human astrocytic cells, CCF and INHA cells were reverse transfected with siRNA directed against mRNAs encoding CLU. First, we examined CLU expression by indirect immunofluorescence analysis and observed a decrease in CLU levels in knockdown cells (Fig. 1A). To further assess the efficacy of CLU downregulation, Western blotting analysis was performed using samples of cells transfected with siCLU siRNA or siNC (negative control). Compared with the control group, there was a significant decrease in CLU in both CCF and INHA cells in the siCLU groups (Fig. 1B and C).

The MTT assay showed significantly decreased metabolic activity in the siCLU groups, indicating an effect of CLU downregulation on cell growth (Fig. 1D and Supplementary Fig. S1), which was further confirmed by the crystal violet assay in combination with bright-field imaging (Fig. 1E and F). Importantly, downregulation of CLU was associated with a significant reduction in cell migration of both cell lines tested (Supplementary Fig. S2). Interestingly, the migration rate of INHA cells was more affected by CLU deficiency than that of CCF cells. We observed that the transient siCLU-induced cell growth arrest was maintained only until the fourth day, after which cell growth resumed by the sixth day (Supplementary Fig. S3). To investigate the effects of CLU downregulation on Ki-67 protein, a key marker for mitotic division, we quantified Ki-67 immunofluorescence in the nucleus using Olympus ScanR imaging. Our results demonstrated a significant reduction of Ki-67 expression in siCLU cells compared to siNC cells, indicating a cell cycle arrest (Fig. 1G and 1H, Supplementary Fig. S4). The development of growth arrest in astrocytes three days after transfection of cells with siCLU was also monitored by time-lapse microscopy using the CytoSMART™ Lux2 Duo Kit platform (CytoSMART™ Technologies B.V., Eindhoven, the Netherlands) (Supplementary video). These findings indicate a loss of cell proliferation in astrocytes when CLU is downregulated, suggesting a crucial role of CLU in cell cycle regulation.

### Downregulation of CLU Induces DNA Damage and Cellular Senescence in Astrocytes

**Fig. 2:**
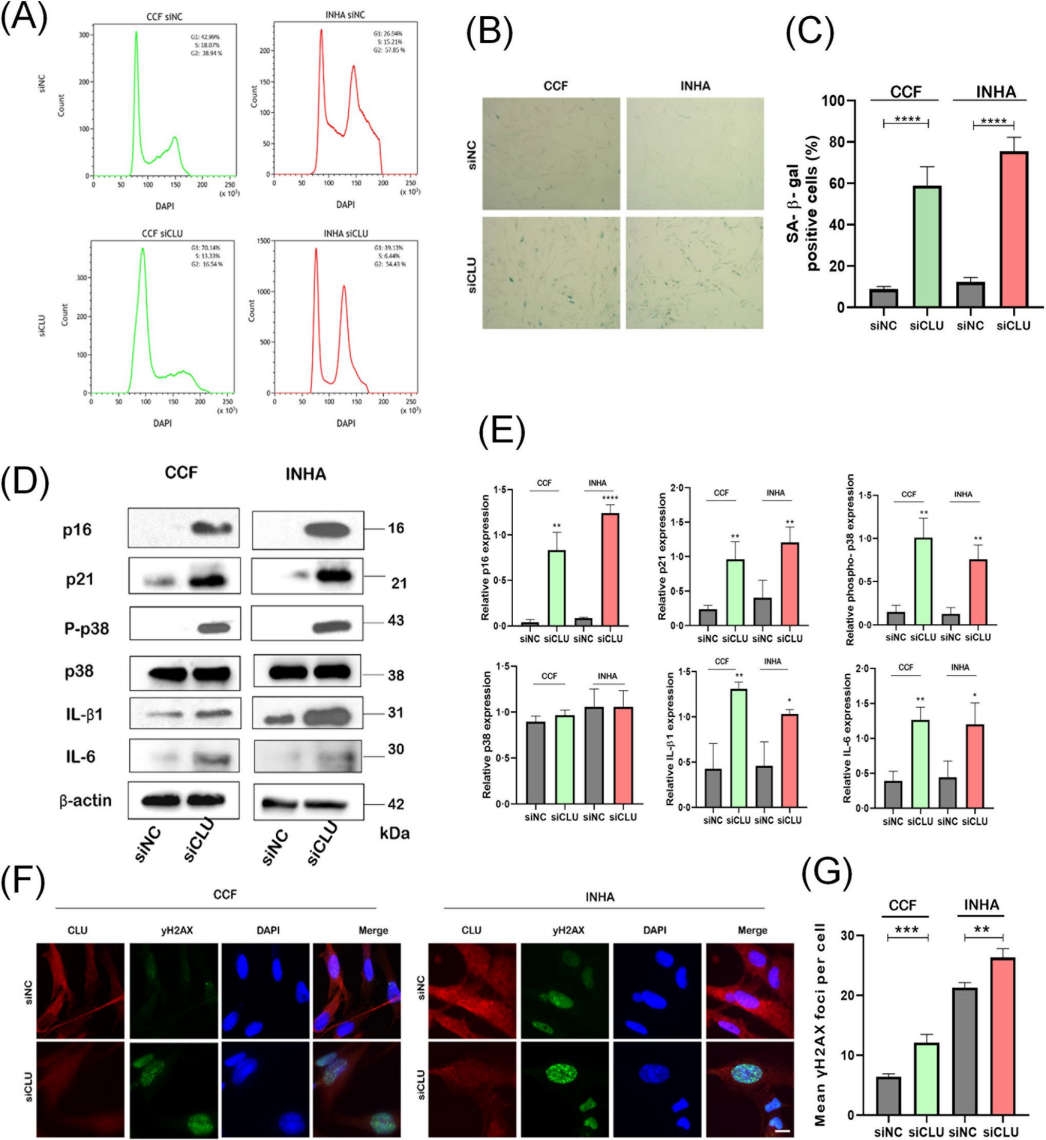
Downregulation of CLU induces cellular senescence by damaging DNA. Flow cytometric analysis of cell cycle phases in CCF and INHA cells shows a G1 arrest in both cell lines after siCLU treatment (**A**). Visualization of astrocyte senescence by SA-β-gal staining in control (siNC) and CLU-deficient (siCLU) cells (**B**). Quantitative evaluation of positively stained blue cells indicates a significantly higher number in the siCLU group compared with the siNC group (**C**). Representative Western blots show the expression levels of proteins associated with senescence and their respective signaling pathways in siNC and siCLU cells (**D**). Quantitative analysis of the expression of p16, p21, p38, phospho-p38 (P-p38), IL-beta1, and IL-6 using Western blotting (**E**). Immunofluorescence imaging (40 × magnification) of gammaH2AX foci in siNC and siCLU cells (**F**) and their quantification with the Olympus ScanR screening system (**G**). Values represent the mean ± SEM of three independent experiments (*p < 0.05; **p < 0.01; ***p < 0.001, ****p < 0.0001)

To investigate the mechanism of cell cycle arrest, we first performed cell cycle analysis by flow cytometry (FACS). The proportion of cells in the G1 phase increased significantly, accompanied by a simultaneous decrease in the S phase in both cell lines tested (Fig. 2A), indicating a suppression of cell cycle progression and a blockage of the cell cycle at the G1/S phase boundary. Downregulation of CLU resulted in morphological changes characteristic of cellular senescence, with a typical upregulation of senescence-associated beta-galactosidase activity by 59% in CCF cells and 78% in INHA cells (Fig. 2B and C). Moreover, the level of cyclin-dependent kinase inhibitor p21 increased approximately twofold in CCF and INHA cells after CLU knockdown (Fig. 2D and E), indicating activation of cell cycle checkpoints. To investigate the mechanism of p21 activation, we first examined the presence of DNA double-strand breaks (DSBs) by staining the DNA damage foci (Fig. 2F and G, and Supplementary Fig. S5). In contrast to the CCF and INHA control cells, our quantification revealed an increase in cells that exhibited significant positivity for γH2AX-foci after 72 h of CLU downregulation. This observation suggests that the activation of the G1/S checkpoint may be attributed, at least in part, to the induction of the DNA damage response (DDR). In a further attempt to elucidate the effects of CLU downregulation on the cell cycle, we observed an increase in Cdk inhibitor p16 protein levels in association with higher levels of phosphorylated p38MAPK in the siCLU groups (Fig. 2D), suggesting an increase in oxidative stress.

To further explore the complexity of siCLU-induced senescence, we determined several other markers associated with senescence by Western blotting. Indeed, the pro-inflammatory cytokines IL-6 and IL-B1 were upregulated at the protein level in CLU-suppressed CCF and INHA cells (Fig. 2D and E). Increased level of heat shock protein HSP90 and downregulation of lamin B1 and EAAT1 were also observed in CLU-suppressed CCF and INHA cells (Supplementary Fig. S6), further supporting the development of a senescent phenotype. In summary, these findings indicate that the reduction in CLU expression initiates a cellular response characterized by the onset of cellular senescence in both examined astrocyte cell lines.

### Downregulation of CLU Leads to Increased Formation of ROS

**Fig. 3:**
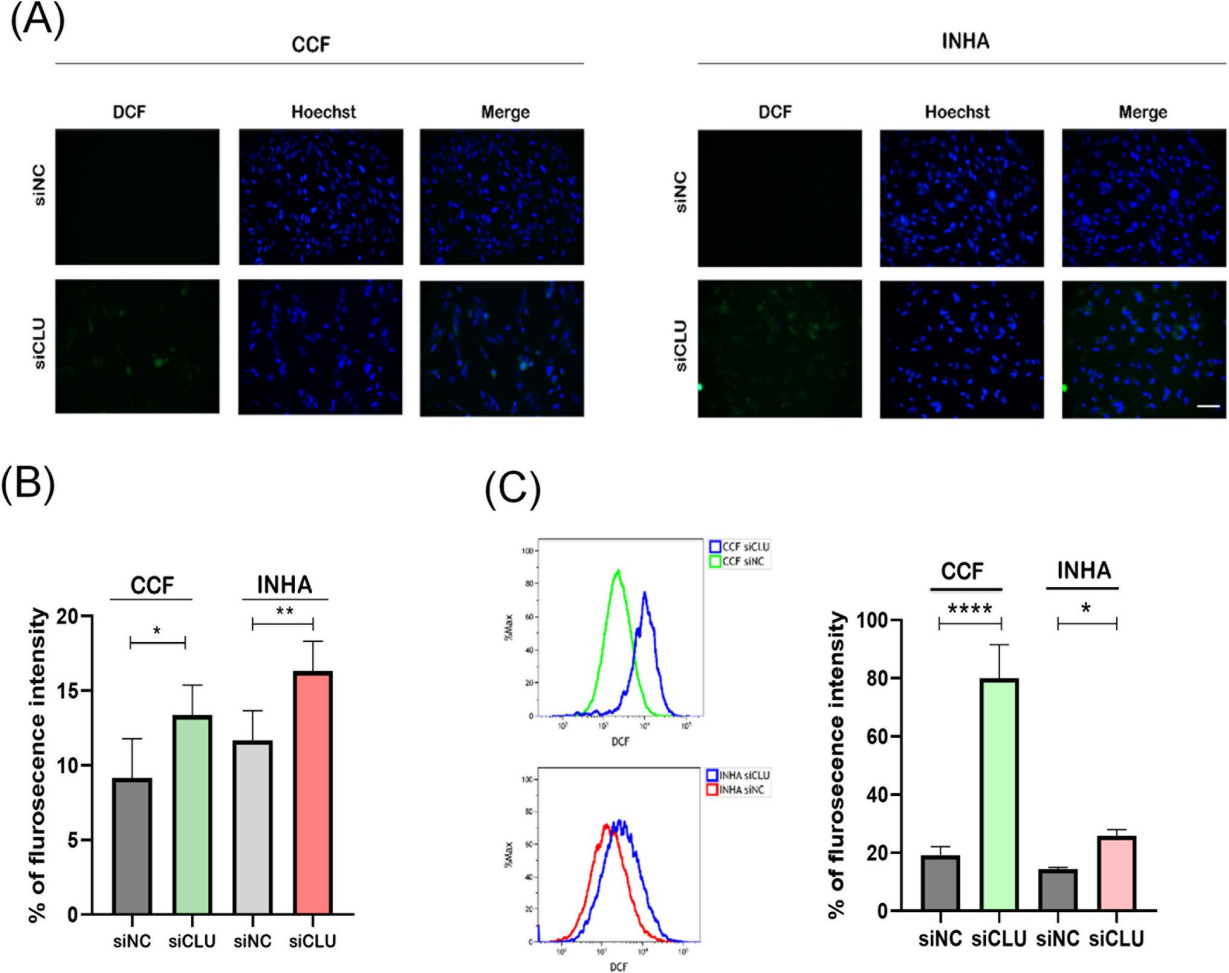
Downregulation of CLU triggers oxidative stress in astrocytes. The relative ROS levels in siNC- and siCLU-treated CCF and INHA cells were assessed using fluorescence microscopy and the DCF-DA (DCF) probe (20 × magnification; scale bar, 200 µm) (**A**). Quantitative analysis shows a significant increase in ROS in CLU-deficient cells compared to controls (**B**). Flow cytometry analysis of siNC and siCLU cells stained with DCF-DA (**C**). Values represent the mean ± SEM of three independent experiments (*p < 0.05; **p < 0.01, ****p < 0.0001)

To investigate whether CLU deficiency induces oxidative stress in astrocytes, we next measured changes in reactive oxygen species (ROS) content using the redox-sensitive DCF-DA fluorescence probe. In both cell lines tested, an increase in intracellular ROS levels was observed after CLU downregulation (Fig. 3A). Quantification of fluorescence showed an approximately 20% increase in ROS in siCLU cells (Fig. 3B). Accordingly, flow cytometry analysis also detected increased ROS levels in the siCLU groups compared to the siNC groups (Fig. 3C). We then investigated whether the elevated ROS levels in siCLU cells could be attenuated by administering antioxidants such as N-acetylcysteine (NAC). After 72 h of cell transfection with siCLU, 10 mM NAC was added to the cells to suppress ROS production, and the cells were incubated for 24 h. In both cell lines with downregulated CLU, NAC treatment caused a significant decrease in ROS levels (Supplementary Fig. S7). Quantification of ROS in cells treated with NAC showed a significant attenuation of ROS production caused by the downregulation of CLU. It is worth noting that we did not detect any significant toxicity using the MTT assay even after 24 h of exposure to 10 mM NAC (data not shown). These data suggest that initiating cell cycle arrest in CLU-deficient cells is likely related to ROS-mediated stress responses.

### Downregulation of CLU Alters Mitochondrial Function

**Fig. 4:**
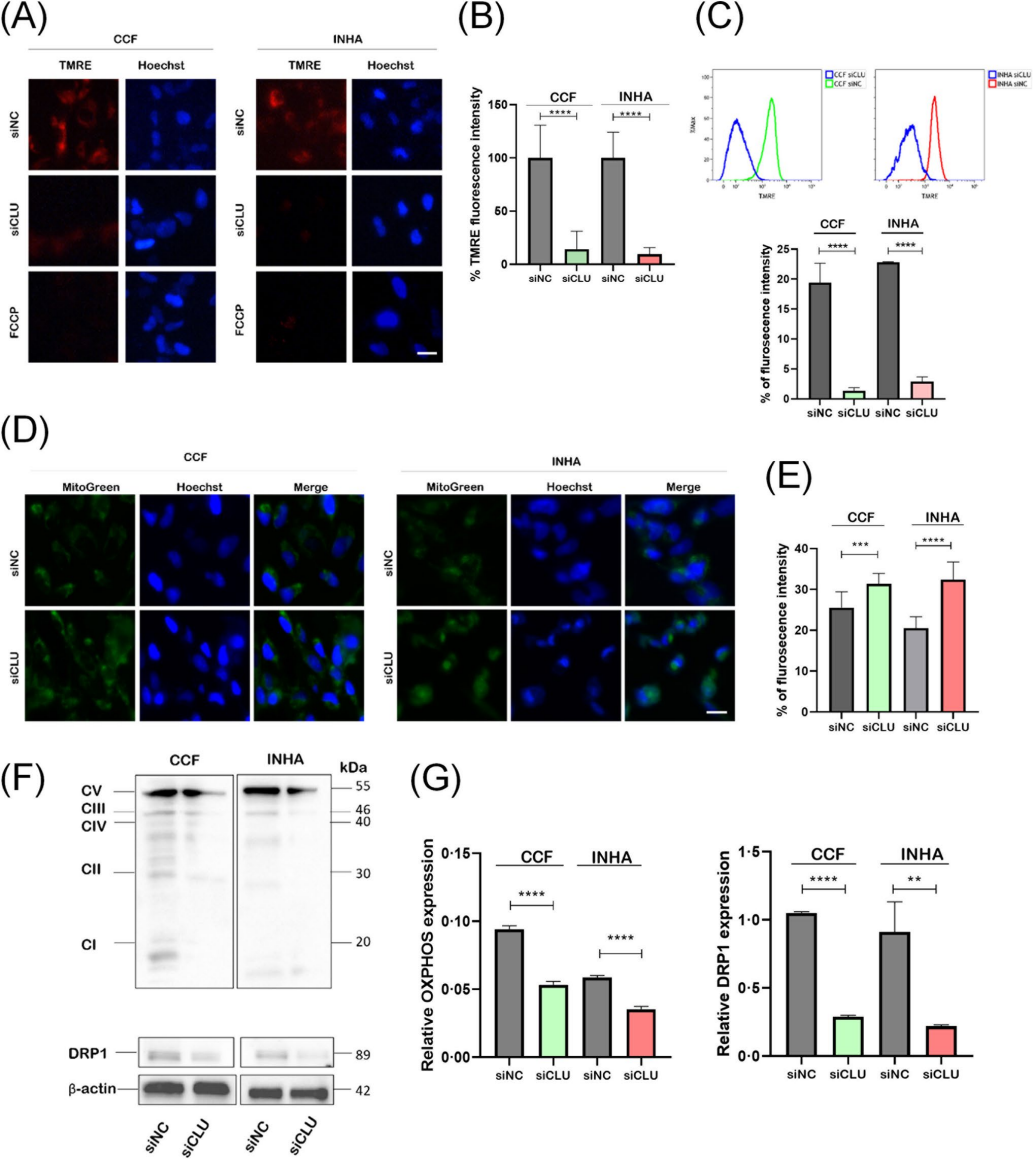
Downregulation of CLU changes mitochondrial functional state. Changes in mitochondrial membrane potential in siNC- and siCLU-treated CCF and INHA cells were visualized using fluorescence microscopy (20 × magnification; scale bar, 50 µm) and the TMRE fluorescent probe (**A**). The fluorescence intensity of TMRE was quantified using a microplate reader and normalized to the Hoechst 33,342 signal (**B**). Flow cytometry analysis of siNC and siCLU cells stained with TMRE (**C**). Mitochondrial mass in individual cell samples was visualized using MitoTracker Green (**D**) and quantified using a microplate reader (**E**). Relative protein expression of OXPHOS complexes and mitochondrial fission protein DRP1 in control and CLU-deficient cells was assessed by Western blotting (**F**) and quantified in both cell types (**G**). Values represent the mean ± SEM of three independent experiments (**p < 0.01; ***p < 0.001; ****p < 0.0001)

The mitochondria are considered to be the primary organelle responsible for the production of ROS. Here, we sought to determine the possible involvement of mitochondria in the development of the ROS imbalance observed in siCLU cells. First, we examined the mitochondrial membrane potential (ΔΨm) using TMRE; the mitochondrial ΔΨm uncoupler FCCP was used as a control. We observed a severe loss of ΔΨm in siCLU cells compared to siNC cells (Fig. 4A). Quantitatively, siCLU cells exhibited a significant decrease in membrane potential (by about 80%) (Fig. 4B), indicating a decline in mitochondrial membrane function. Flow cytometry confirmed these findings and showed consistent results (Fig. 4C). Subsequent analysis of ATP production revealed a decrease in the total ATP production rate and a higher glycolytic than oxidative (mitochondrial) ATP production rate in CLU-deficient CCF and INHA cells (Supplementary Fig. S8). These results suggest that downregulation of CLU promotes a glycolytic phenotype rather than an oxidative phenotype. NAC was then used at a concentration of 10 mM to test whether it could prevent ΔΨm loss. NAC exhibited a modest compensatory effect on the decrease in ΔΨm caused by CLU downregulation in both cell types. This was reflected in the increase in TMRE fluorescence intensity, which was approximately 17% higher in CCF cells and 35% higher in INHA siCLU cells than in the siCLU group without NAC treatment (Supplementary Fig. S9). These observations are consistent with previous studies conducted with NAC and its prodrugs and emphasize their potential to attenuate changes in mitochondrial membrane potential [30].

Some studies have shown higher mitochondrial mass in replicative and oxidative stress-induced senescence in fibroblasts [31]. To confirm the presence of impaired mitochondria in siCLU cells, we performed a mitochondrial mass assessment using MitoTracker Green. Our fluorescence image analysis revealed a marked increase in mitochondrial mass in siCLU cells (Fig. 4D). Quantification of changes in fluorescence signal intensity revealed a statistically significant increase in MitoTracker Green signal in CCF (by 11%) and INHA (by 59%) cells (Fig. 4E). This observation suggests a compromised quality control mechanism within the mitochondrial system, leading to an accumulation of dysfunctional mitochondria.

Next, we performed a Western blotting analysis to determine the expression levels of the OXPHOS complexes (complex I, II, III, IV, and V). Downregulation of CLU was associated with a statistically significant suppression of protein levels of complex I, II, III, and IV, while complex V was not significantly altered (Fig. 4F and G). We also found a strongly decreased expression (by about 75%) of the mitochondrial fission protein DRP-1 (Fig. 4F and G), possibly due to prolonged mitochondrial stress or damage in both cell lines tested. The cells may respond by downregulating DRP1 as a protective mechanism to prevent excessive fission, which could lead to mitochondrial fragmentation and dysfunction [32]. These results suggest that the senescence phenotype induced by the downregulation of CLU is associated with the alteration of mitochondrial function.

## Discussion

CLU is a versatile protein implicated in DNA repair, cell cycle regulation, apoptotic cell death, and tumorigenesis. It is often overexpressed in solid tumors, conferring a prosurvival advantage against treatments [33]. In the present study, we investigated the consequences of suppressing CLU gene transcription in two human astrocytic cell lines, CCF-STTG1 and INHA. Our observations revealed that downregulation of CLU leads to a marked decrease in cell proliferation rate in both tested types of astrocytes. Moreover, our results showed that the reduction in proliferation may be linked to changes typical for the onset of cellular senescence. This study highlights a potential interplay between CLU, cell proliferation, and senescence in astrocytes, shedding light on the role of CLU in these cellular processes. Furthermore, the reduction in cell migration rate observed after transfection with siCLU suggests a crucial involvement of CLU in astrocyte migration. This observation is in consistent with a previous finding that CLU promotes chemotactic migration of macrophages [34].

One of the primary stress sources provoking cellular senescence is unhealed DNA damage, which triggers persistent activation of the DDR, leading to activation of the ATM/p53/p21WAF1 pathway [35], where p21WAF1 inhibits the cell cycle regulatory cyclin-dependent kinases Cdk4 and Cdk2 [36]. Here, we observed increased expression of senescence markers p16INK4A and p21WAF1, indicating cell cycle arrest in response to CLU downregulation. DNA damage caused by radiation, chemical agents, or the accumulation of ROS is known to be the most important factor triggering cellular senescence-related stress [37]. Our present study revealed a significantly increased number of DNA damage foci marked with phosphorylated histone H2AX in cells with downregulated CLU, indicating that DSBs are a secondary consequence of CLU downregulation and that CLU deficiency may lead to a failure of the DNA damage repair system.

Several factors have been shown to disrupt the balance of redox homeostasis, leading to DNA damage. Previous research has demonstrated that activation of p38 (MAPK14) can stimulate the production of ROS in cells, involving both mitochondrial and non-mitochondrial (NADPH oxidase-dependent) pathways [38]. This response also appears to be the case in our experimental model, as we found markedly activated p38MAPK and increased ROS levels in CLU-deficient astrocytes, resulting in DNA damage in these cells. It is worth noting that the induction of cellular senescence by siCLU may involve distinct mechanisms in different cell types. For example, Mitsufuji et al. reported that downregulation of CLU in pancreatic cancer cells did not result in significant alterations in the phosphorylation of p38 kinase [21]. This observation suggests that there are still gaps in our understanding of the precise mechanisms by which CLU deficiency leads to cell cycle arrest. Further research is needed to explore these mechanisms more comprehensively. In many cases, increased ROS production is due to unstable homeostasis and altered mitochondrial functions. The involvement of mitochondrial ROS in the maintenance of cellular senescence through a self-reinforcing loop that perpetually induces DNA damage and causes the senescent phenotype is becoming apparent [39].

Astrocytes have been extensively studied in the context of oxidative stress triggers, such as H_2_O_2_, which can induce premature senescence. While the effects of mitochondrial ROS on senescence is well documented in cultured cells, their importance in the *in vivo* context is still less known. Nevertheless, studies performed *in vivo* with models of mitochondrial dysfunction and increased oxidative stress show an enhancement of senescence in various tissues. For example, when the mitochondrial enzyme monoamine oxidase A is specifically overexpressed in cardiomyocytes, a phenomenon is observed in cardiac aging that leads to an increase in ROS and subsequent mitochondrial dysfunction [40]. Mitochondrial alterations have a major impact on cellular redox imbalance and contribute to cellular senescence. In our study, CLU deficiency in astrocytes led to increased intracellular ROS levels. The mechanism underlying siCLU-induced senescence prominently points to a disturbance of the ROS balance, which could be one of the driving factors for triggering cellular senescence. Importantly, the elevated ROS levels could be partially reduced by treating the cells with the antioxidant NAC, which also restored mitochondrial potential. Therefore, CLU is an important factor in maintaining the redox balance.

In senescent cells, ATP production in mitochondria decreases, possibly due to reduced OXPHOS efficiency caused by a decline in mitochondrial membrane potential and reduced H^+^ concentration in the intermembrane space [39]. Normally, damaged mitochondria can fuse with healthy ones or undergo fission to maintain cellular health. However, senescence is associated with altered mitochondrial dynamics, characterized by elongated and hyperfused mitochondria due to impaired mitochondrial fission protein DRP-1 [41]. In our study, we found dynamic changes in the functional state of mitochondria in CLU-deficient cells, reflected in a pronounced reduction of complexes I, II, III and IV, a lower mitochondrial membrane potential, a reduced mitochondrial fission rate, a reduced ATP production rate, and the transition to a glycolytic phenotype. These data suggest that the downregulation of CLU contributes significantly to mitochondrial dysfunction.

One hallmark of the senescent phenotype is the production of pro-inflammatory cytokines, including IL-6 and IL-B1, as part of the SASP [42]. The secretion of pro-inflammatory chemokines, cytokines, proteases, and growth factors can be regulated by NF-κB [43]. Here, we detected increased levels of IL-6 and IL-B1, which, along with decreased levels of EAAT1 and lamin B1, support the development of a senescent phenotype in CLU-deficient cells. It has been reported that ginsenosides can prevent, but not reverse, H₂O₂-induced senescence in astrocytes and astroglioma cells, and that they partially prevent oxidative stress-induced reduction of proinflammatory SASP components, suggesting that some treatments may attenuate the adverse consequences associated with senescence [44]. Interestingly, astrocytes surrounding amyloid-beta (Aβ) plaques were also found to have higher levels of IL-6 [45].

Dysregulation of CLU has been linked to various pathological conditions, including neurodegenerative diseases and cancer [46]. In neurodegenerative disorders such as Alzheimer’s disease (AD), CLU levels are elevated, particularly near Aβ plaques. CLU interacts with Aβ, influencing its aggregation and clearance. Dysregulated CLU impairs Aβ clearance, leading to the formation of toxic aggregates and worsening the progression of AD [47]. CLU also plays a role in other neurodegenerative diseases such as Parkinson’s disease and ALS. It helps to respond to cellular stress and maintain neuronal health. Dysregulation of CLU disrupts its protective function, increasing neuronal vulnerability and disease progression [46]. On the other hand, CLU plays a complex role in cancer, exhibiting both tumor-promoting [48] and tumor-suppressing [49] properties, depending on the type of cancer. Dysregulation of CLU can occur at the level of gene expression (epigenetic regulation) and post-translational modification. For example, the GC-rich region with a methylated CpG island located just upstream of the 5′ end of the CLU promoter suggests that DNA methylation regulates CLU gene expression [50]. The complexity of CLU dysregulation has significant implications for human health. Investigating how CLU dysregulation occurs could lead to new therapeutic approaches targeting this versatile protein. It is plausible that inducing senescence by silencing CLU, in combination with senolytics, could help eliminate of tumor recurrence.

In summary, the results of this study demonstrate that suppression of CLU in astrocytes effectively induces senescence by altering mitochondrial function and increasing ROS production and DNA damage. We have shown that downregulation of CLU leads to suppression of cell proliferation in both human astrocyte cell lines tested. These findings suggest that targeting CLU may be a promising therapeutic approach for treating gliomas. In this context, it is worth mentioning that CLU was identified as one of the hundred most upregulated marker proteins in glioblastoma tumor tissues [51]. By inducing cellular senescence through CLU silencing, this treatment strategy could suppress glioma cell growth and improve prognosis and outcomes for glioma patients. Due to its potential therapeutic significance, the role of CLU in astrocyte senescence and function deserves further investigation.

## Supplementary Information

Below is the link to the electronic supplementary material.

Supplementary file1 (AVI 93477 KB)

Supplementary file2 (AVI 92883 KB)

Supplementary file3 (AVI 89577 KB)

Supplementary file4 (AVI 81377 KB)

Supplementary file5 (PDF 1676 KB)

Supplementary file6 (PDF 436 KB)

## Data Availability

No datasets were generated or analysed during the current study.
