# Supplementary material for: Clusterin Deficiency Promotes Cellular Senescence in Human Astrocytes
Source: Mol Neurobiol. 2024 Dec 3;62(5):5774–86. doi: 10.1007/s12035-024-04650-2 (PMC11953114; doi:10.1007/s12035-024-04650-2)
Supplement: Supplementary file 5 — Supplementary file5 (PDF 1676 KB) [file 12035_2024_4650_MOESM5_ESM.pdf]

Supplementary Figures

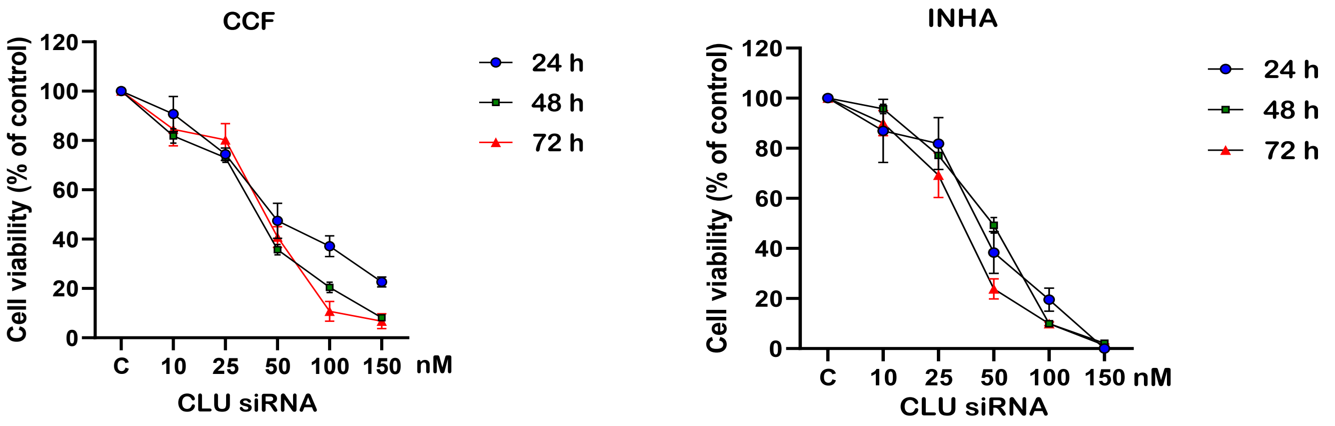

Supplementary Figure S1. Effect of CLU downregulation on cell proliferation of human astrocytes. CCF and INHA cells were transfected with different concentrations of CLU siRNA (siCLU) and cell proliferation was monitored for up to 72 hours using the MTT assay. siCLU at a concentration of 25 nM was used in all subsequent transfection experiments.

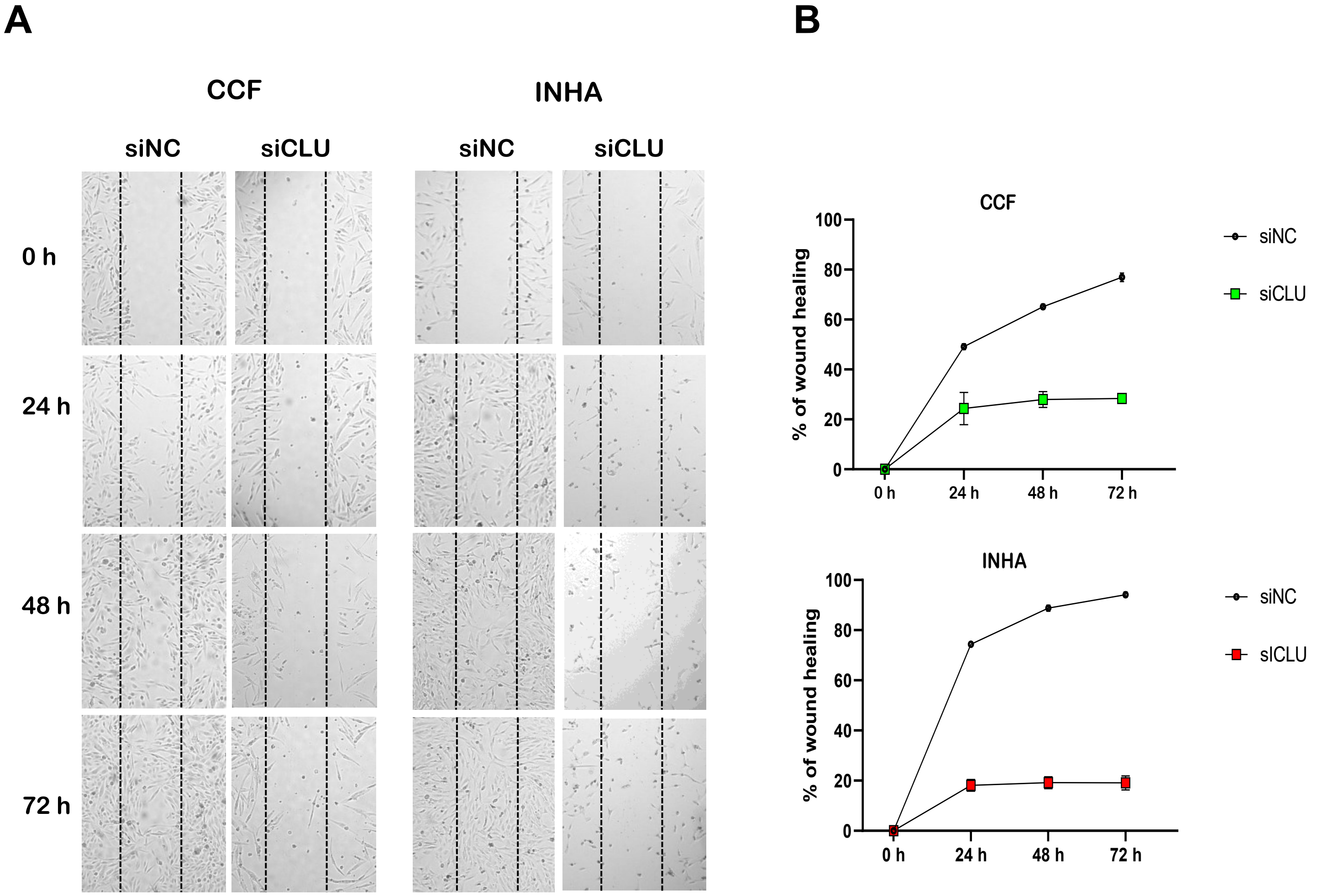

Supplementary Figure S2. Scratch wound healing assay. Once the cell layers of CCF and INHA cells transfected with siNC or siCLU were confluent, they were scraped in a straight line with a 1-mm pipette tip. Images of the cell cultures were then taken every 24 hours using a phase contrast microscope at 4× magnification (A). The width of the scratch wound was evaluated with ImageJ. Wound healing, which reflects the rate of cell migration, was determined from three individual experiments and expressed as a percentage (B). Values represent the mean ± SEM of three independent experiments.

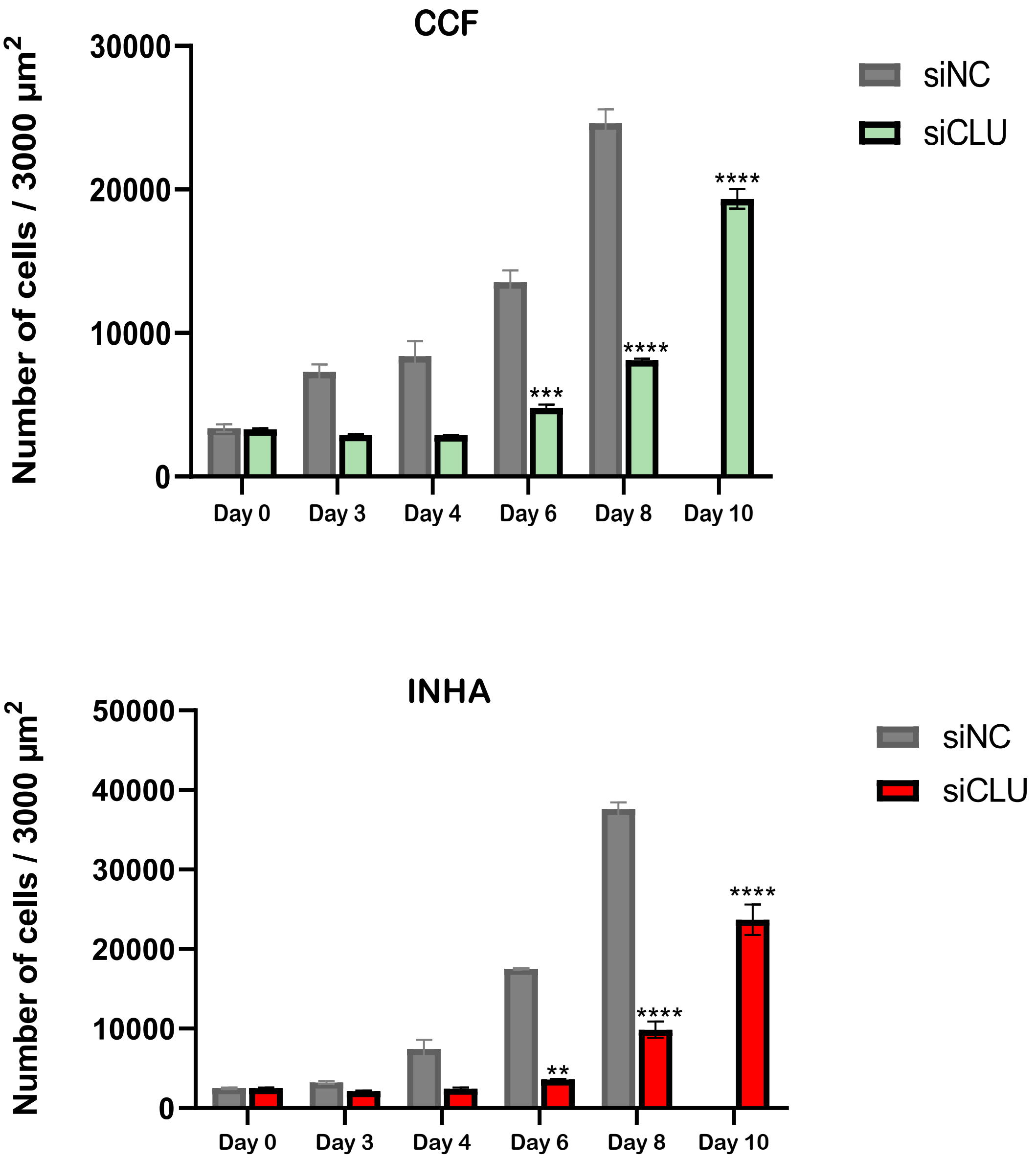

Supplementary Figure S3. Effect of transient siCLU transfection on cell number during prolonged cultivation of CCF and INHA cells. Automated cell counting was performed using the BioTek Cytation 5 cell imaging multimode reader (Agilent). The number of control cells (siNC) is not shown for day 10, as the cells were heavily overgrown after day 8. The growth arrest of cells transiently transfected with siCLU lasted until day 4. Values represent the mean ± SEM of three independent experiments (**$p < 0.01$; ***$p < 0.001$; ****$p < 0.0001$ *vs* day 0).

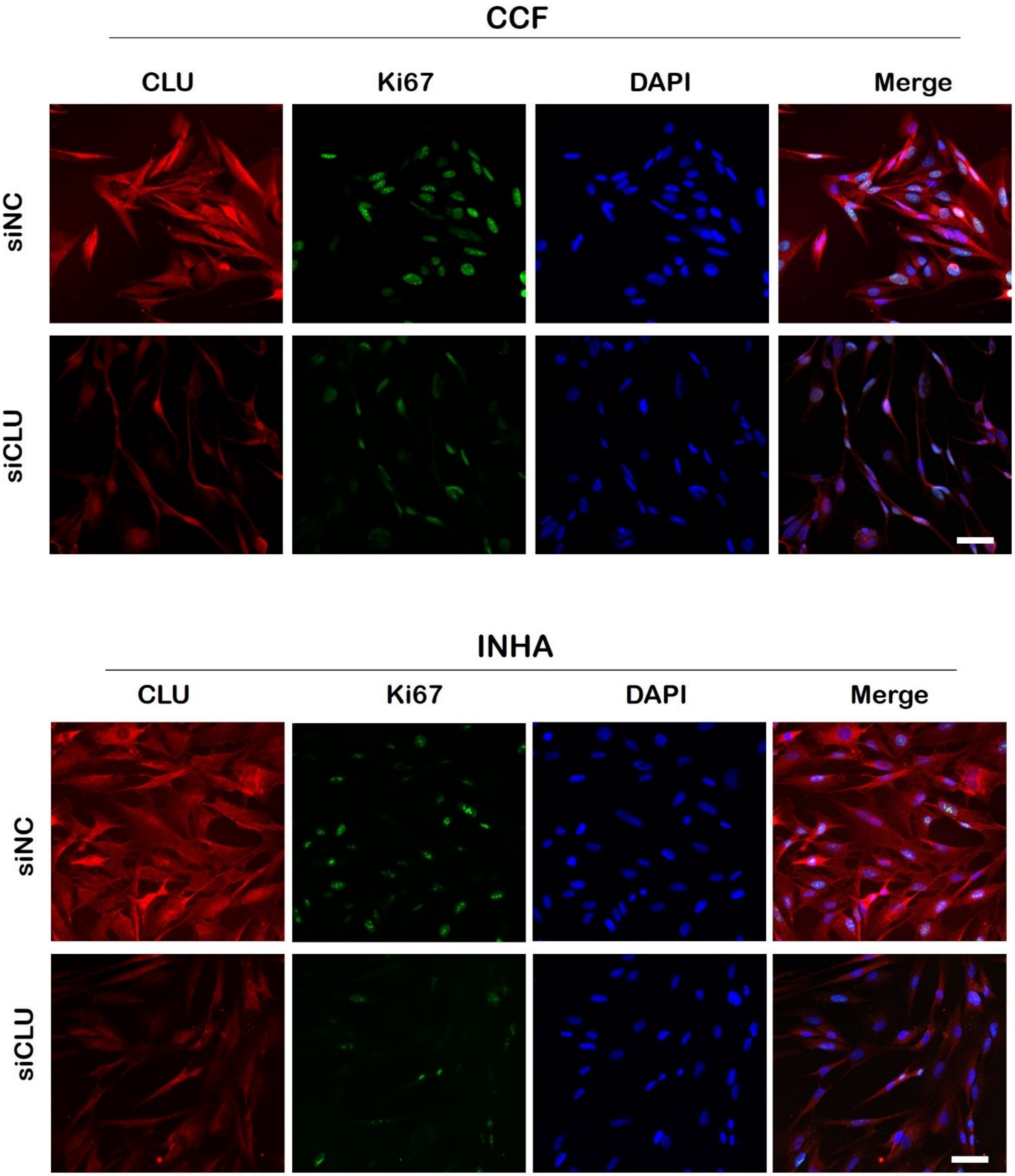

Supplementary Figure S4. Immunofluorescence microscopic imaging of proliferative capacity in control (siNC) and CLU-deficient (siCLU) CCF and INHA cells. Ki67 bodies in these cells were inspected and counted using a 40× objective. Scale bar 50 µm.

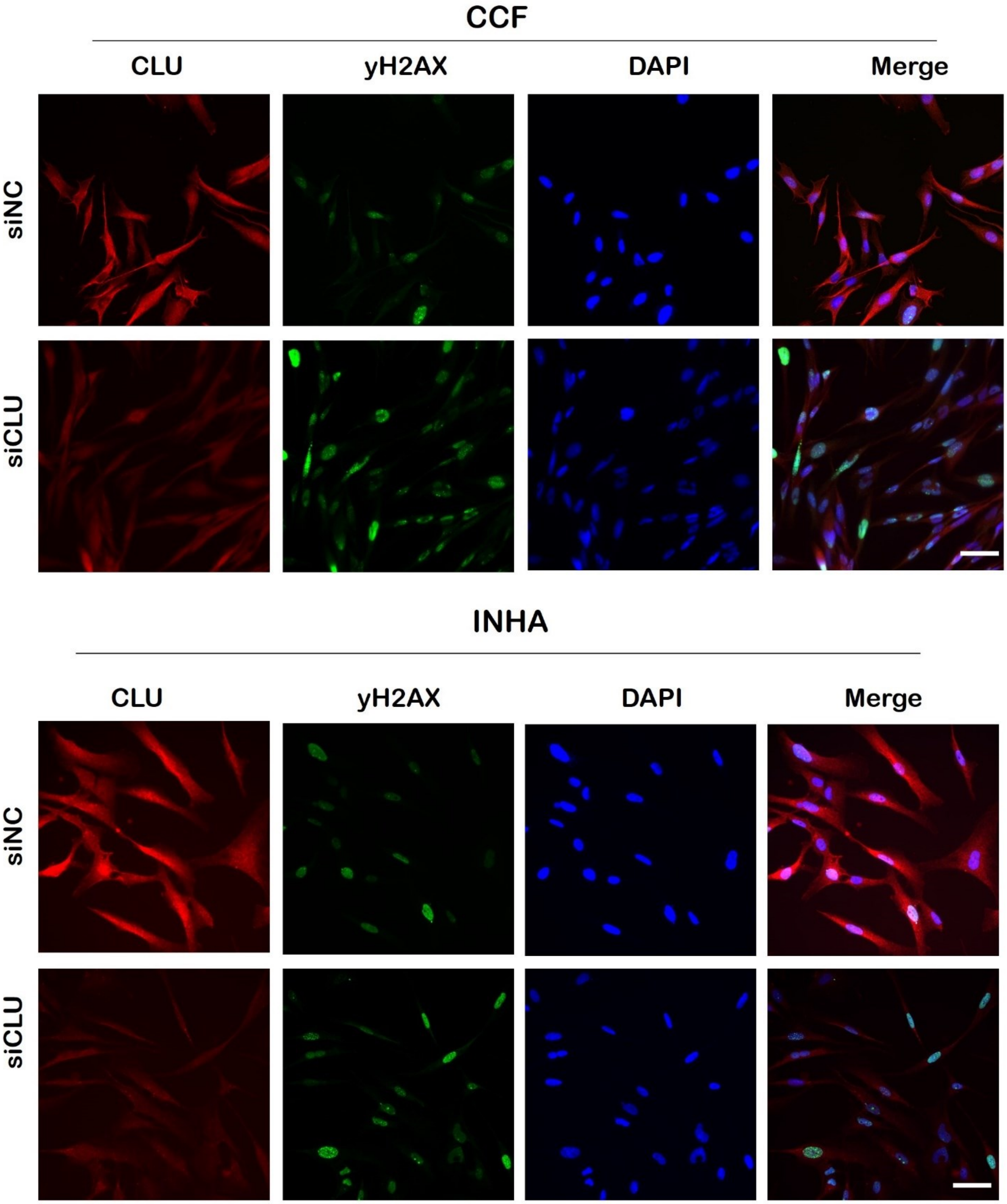

Supplementary Figure S5. Immunofluorescence microscopic imaging of DNA damage foci in control (siNC) and CLU-deficient (siCLU) CCF and INHA cells. yH2AX foci in these cells were inspected and counted using a 40× objective. Scale bar 50 μm.

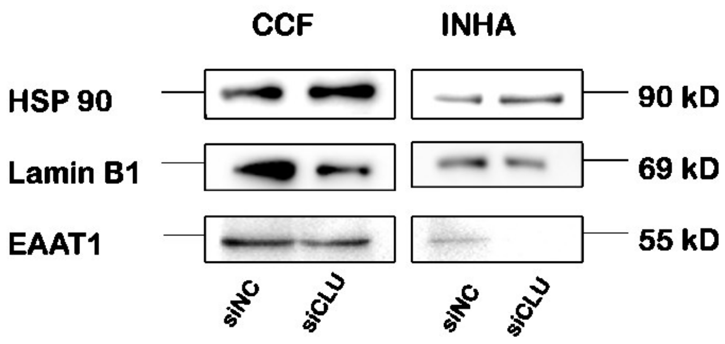

Supplementary Figure S6. The effect of CLU downregulation on the expression levels of selected SASP markers in CCF and INHA cells. Representative examples of Western blots show upregulation of HSP90 downregulation of lamin B1 and EAAT1 (Excitatory amino acid transporter 1) in siCLU-treated CCF and INHA cells.

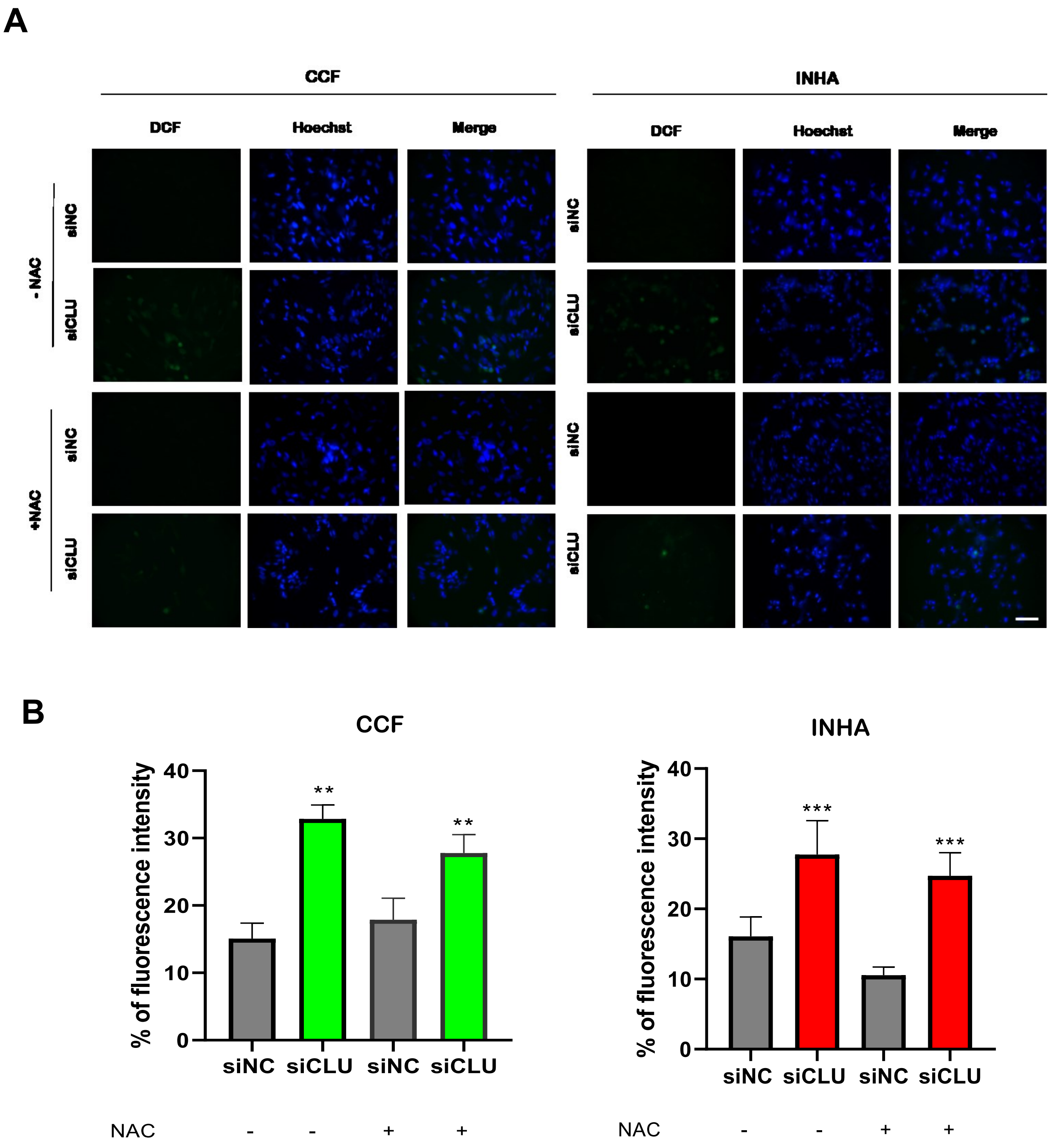

Supplementary Figure S7. Attenuation of oxidative stress by N-acetylcysteine in CLU-deficient CCF and INHA cells. Fluorescence images show detection of ROS in CCF and INHA astrocytes treated with siNC (control) or siCLU followed by addition of 10 mM NAC for 24 hours (A). Fluorescence intensity of the DCF probe was quantified using a microplate reader (B). Scale bar 200 µm. Values represent the mean ± SEM of three independent experiments (**p < 0.01; ***p < 0.001 vs the corresponding siNC).

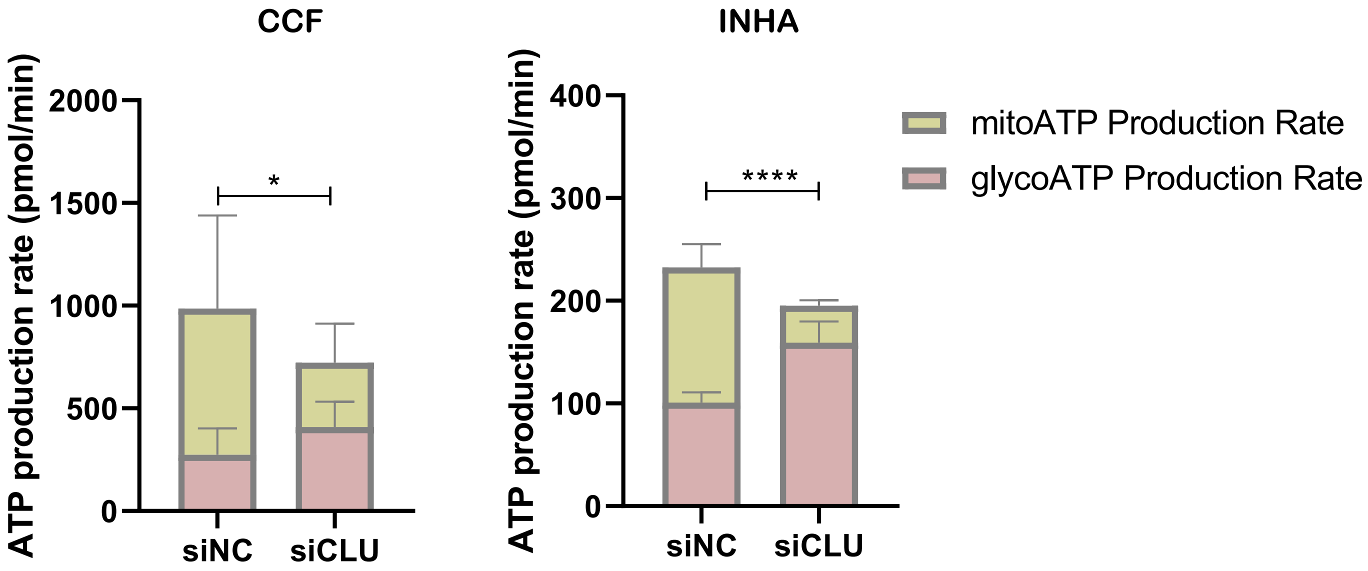

Supplementary Figure S8. Effect of CLU deficiency on ATP production in CCF and INHA cells. ATP production was determined using the Seahorse XF real-time ATP rate assay in siNC-(control) and siCLU-transfected cells. Metabolic flux analysis shows quantification of glycolytic ATP production and mitochondrial ATP production. Values represent the mean ± SEM of three independent experiments (*p < 0.05; ****p < 0.0001).

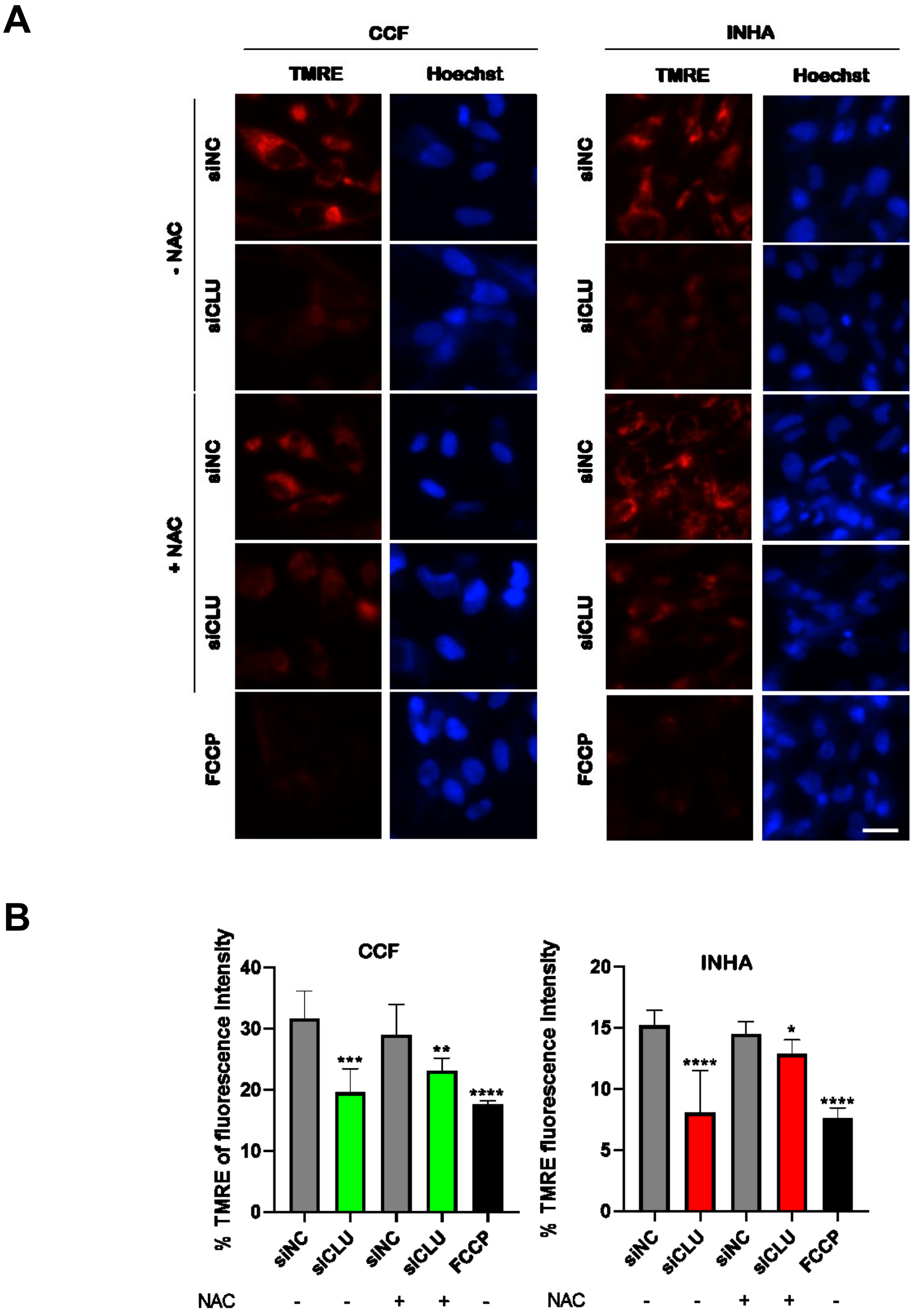

Supplementary Figure S9. Partial restoration of mitochondrial membrane potential by N-acetylcysteine in CLU-deficient human astrocytes. Fluorescence images show detection of mitochondrial membrane potential in CCF and INHA astrocytes treated with siNC or siCLU followed by addition of 10 mM NAC for 24 hours (A). Fluorescence intensity of the TMRE probe was quantified using a microplate reader (B). Sacle bar 50 µm. Values represent the mean ± SEM of three independent experiments (*p < 0.1; **p < 0.01; ***p < 0.001; ****p < 0.0001 *vs* the corresponding siNC).
