# Supplementary material for: Clusterin Deficiency Promotes Cellular Senescence in Human Astrocytes
Source: Mol Neurobiol. 2024 Dec 3;62(5):5774–86. doi: 10.1007/s12035-024-04650-2 (PMC11953114; doi:10.1007/s12035-024-04650-2)

Supplementary Material – Images of the uncropped blots and the gels stained with Ponceau S

Examples of original images of immunoblots and Ponceau S staining. After treatment of CCF and INHA astrocytes with siRNA against CLU (siCLU) or scrambled siRNA (siNC) for 72 hours, cell homogenates were subjected to SDS-PAGE, transferred to a nitrocellulose membrane, stained with Ponceau S (B), followed by blotting with the indicated antibody (A).

I. Antibody against p16

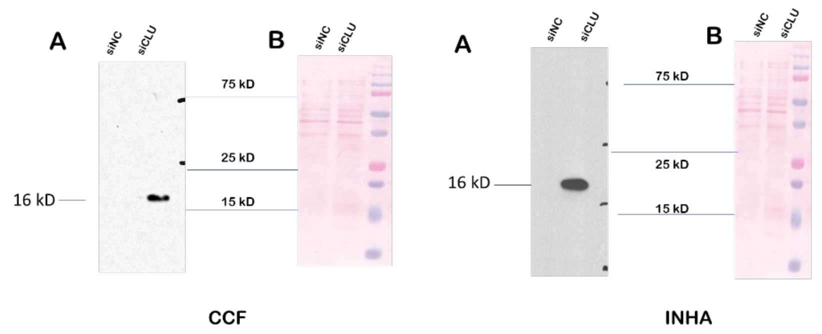

II. Antibody against p21

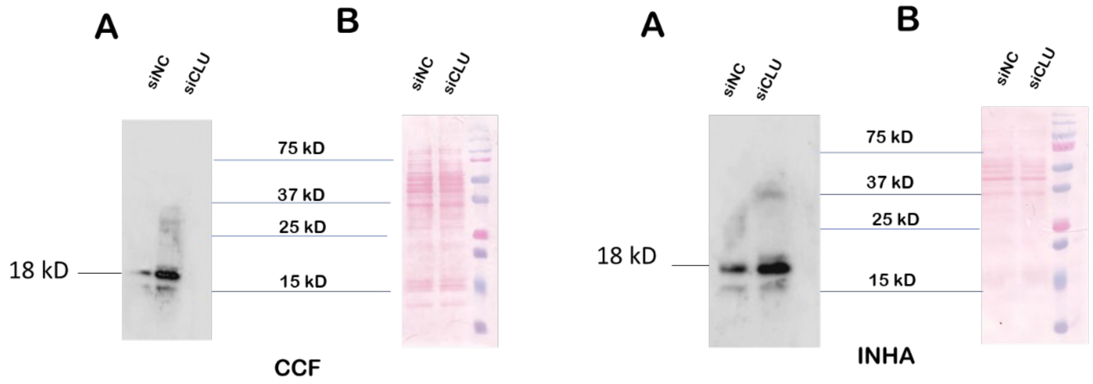

III. Antibody against IL-b1

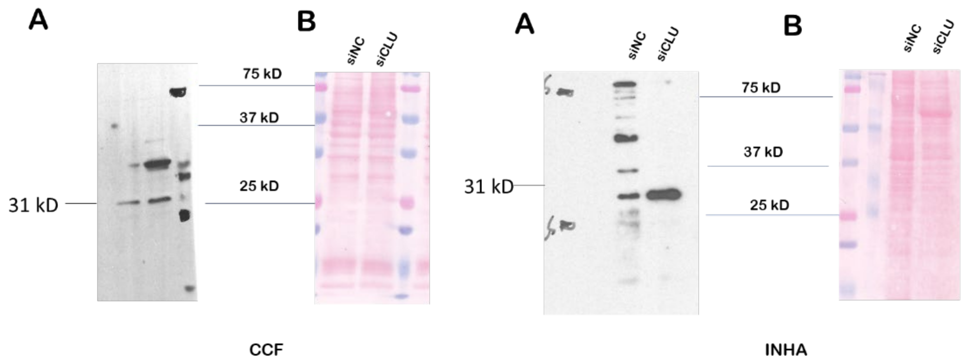

IV. Antibody against phospho-p38

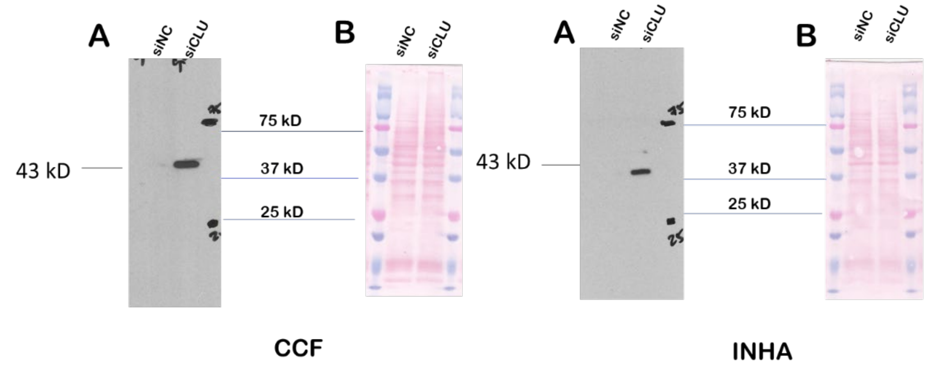

V. Antibody against p38

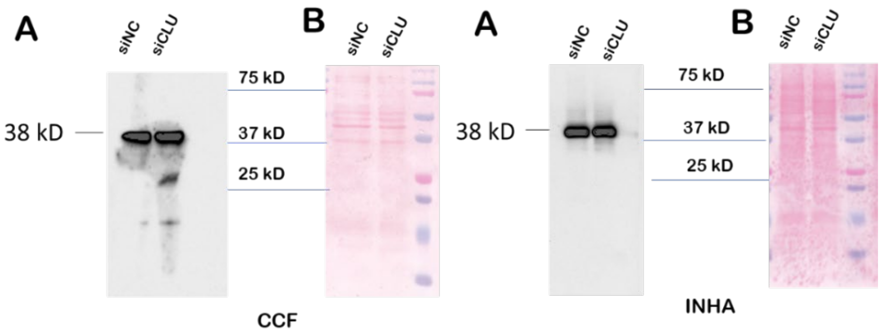

VI. Antibody against IL-6

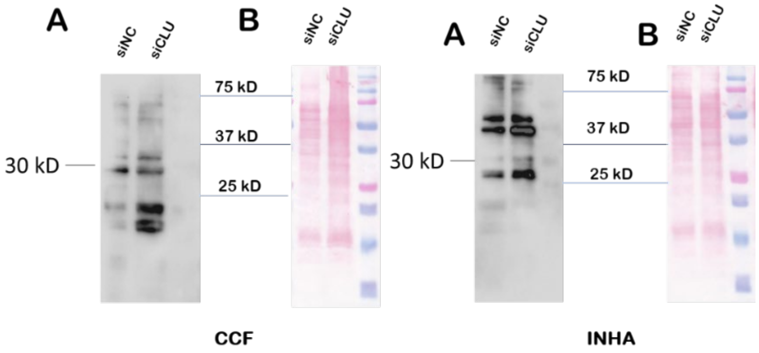

VII. Antibody against OXPHOS complexes

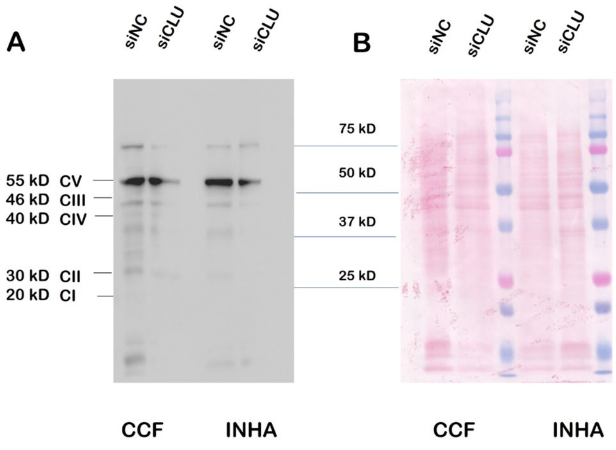

VIII. Antibody against DRP-1

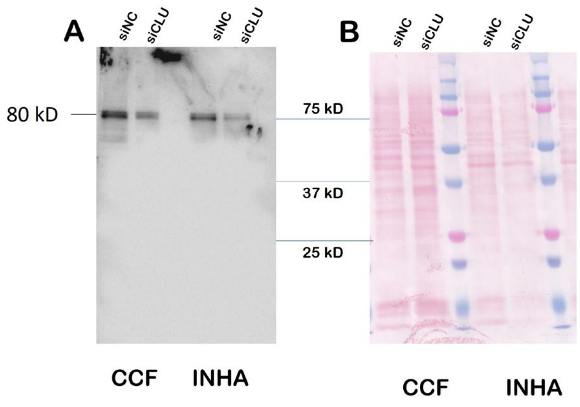

Supplement: Supplementary file 6 — Supplementary file6 (PDF 436 KB) [file 12035_2024_4650_MOESM6_ESM.pdf]
